# Incidence and risk factors for stroke after hip fracture: a meta-analysis

**DOI:** 10.1038/s41598-023-44917-7

**Published:** 2023-10-17

**Authors:** José María Lamo-Espinosa, Gonzalo Mariscal, Jorge Gómez-Álvarez, Mikel San-Julián

**Affiliations:** 1 Hip, Tumors and Pediatric Orthopedic Unit, University Clinic of Navarra, Navarra, Spain; 2 Institute for Research on Muscuoskeletal Disorders, Valencia Catholic University, Carrer de Quevedo, 2, 46001 Valencia, Spain

**Keywords:** Medical research, Risk factors

## Abstract

Hip fractures represent a high burden and are associated with mortality in up to 30% of the cases. Stroke complications can be devastating and increase mortality and disability in elderly patients. This study aimed to determine the overall incidence and risk factors for stroke in patients with hip fractures. A systematic search of the literature using PubMed, EMBASE, Scopus, and Cochrane Collaboration Library databases was carried out. Studies have reported the incidence of stroke in patients > 50 years of age with hip fractures. Data were extracted according to PRISMA guidelines (PROSPERO: CRD42023384742). Data were combined using Review Manager version 5.4. A random-effects model was adopted if a significant heterogeneity was observed. The primary outcome was the incidence of stroke in patients with hip fractures. The secondary outcomes of interest included the influence on the incidence of demographic factors, associated conditions, habits, and analytical parameters. Of the 635 initially retrieved studies, 18 were included, with 256,197 patients. The mean age of the patients ranged from 55 to 84 years old. The overall incidence of stroke in patients with hip fracture was 6.72% (95% CI 4.37–9.07%. The incidence of stroke by region was highest in the American continent (8.09%, 95% CI 3.60–12.58%; P > 0.001). Regarding associated conditions diabetes significantly increased the risk of stroke (OR 1.80, 95% CI 1.41–2.30). Respect to patient characteristics, BMI greater than 24.4 and female gender did not significantly increase the risk of stroke: (OR 1.07, 95% CI 0.74–1.56) and (OR 1.15, 95% CI 0.91–1.46). Lastly, lower albumin concentrations were a risk factor for stroke in patients with hip fracture (MD − 3.18, 95% CI − 4.06 to 2.31). In conclusion, the incidence of stroke after hip fracture was 6.72%. The incidence of stroke increases over time, and the closely associated risk factors are diabetes and low albumin level.

## Introduction

Hip fracture is one of the most frequent fractures and its incidence is expected to increase in the coming years^1^. It represents a high burden and is associated with morbidity and mortality in up to 30% of the cases^2^. A meta-analysis analyzed the incidence of hip fracture after stroke; however, to our knowledge, no meta-analysis has analyzed the overall incidence of stroke after hip fracture^3^. Hip fractures are associated with prothrombotic factors, including bed rest and decreased movement. These complications are related to treatment and hospital stay and are typical of elderly patients^4^. This association varies and remains unclear because of the inconclusive findings of previously published studies^3,5^. The complications of stroke can be devastating, increasing mortality and disability in elderly patient^6^. Moreover, after the COVID-19 pandemic, the mortality and morbidity rates of these pathologies have increased^7^. The mechanism by which hip surgery may increase the risk of thromboembolic events is fatty microembolism, blood loss with vasoconstriction, or hypotension caused by anesthetic drugs. In addition, the use of cement increases thromboembolic risk^8^. Other well-established risk factors include inactivity, weight loss, and alcohol, among others^9^. However, these elderly patients also consume drugs that increase the risk of fragility, osteoporosis, and subsequent hip fractures, such as anxiolytics, antidepressants, antiparkinsonians, or benzodiazepines^9^.

One of the main issues regarding studies analyzing the incidence of stroke after hip fracture is the difference in follow-up periods and the lack of evidence on risk factors. It is essential to know the parameters that determine the risk of stroke in hip fractures, and they should be considered to control them effectively. Studies dealing with this issue highlight the importance of considering which factors are associated with increased risk^10–13^.

The main objective of this study was to determine the overall incidence of stroke in patients with hip fracture. In addition, we aimed to determine which factors influence the incidence and to analyze demographic or secondary variables that may influence the results.

## Material and methods

### Eligibility criteria

**Figure 1:**
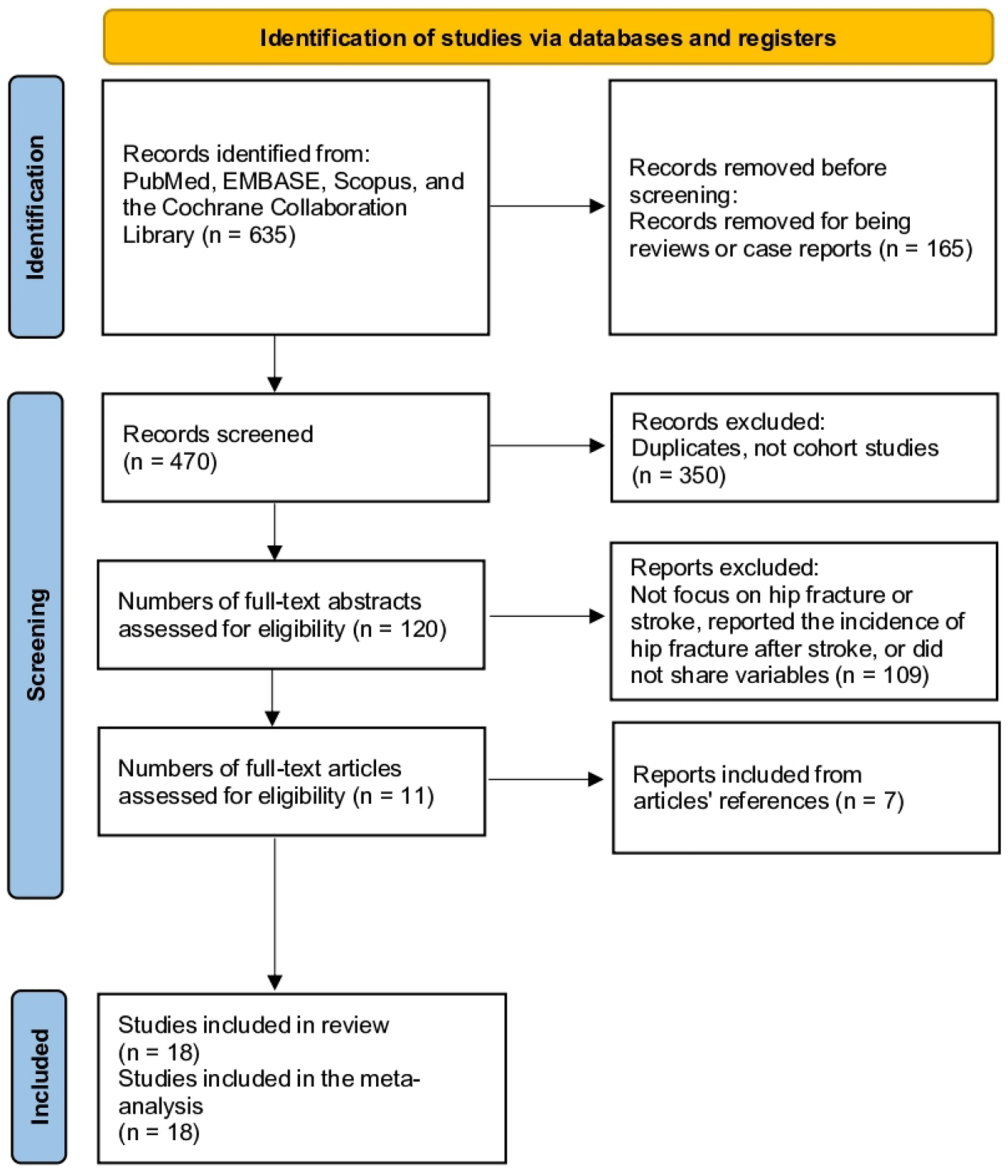
Study selection flow diagram (preferred reporting items for systematic reviews and meta-analysis).

This study was registered with the protocol in PROSPERO (CRD42023384742). The present study followed the PRISMA guidelines (Fig. 1)^14^. The included studies are summarized in Table 1. Studies were considered for inclusion if they met the following predefined criteria: the study must be conducted in a hospital or community setting and enrolling adult patients aged ≥ 50 years who have suffered a hip fracture regardless of subsequent surgical fixation. Both retrospective and prospective studies have been conducted. The primary outcome of interest was the incidence of ischemic, hemorrhagic, and overall strokes. Studies must provide data on stroke events occurring after an index hip fracture or after admission/operation for a fracture. The included studies needed to evaluate and report on potential risk factors for post-fracture stroke, at a minimum examining demographic factors such as age and sex, but preferably also considering pharmacological factors such as antiplatelet or anticoagulant use, medical history, bibliometric factors such as publication year, and analytical factors such as study size and design. Studies that compared populations with and without post-hip fracture stroke were preferred, but those reporting only post-fracture stroke incidence were also eligible if they examined the associated risk factors. No restrictions were placed on language or publication date. Both short-term and long-term follow-up periods were acceptable as long as stroke incidence was measured from the time of hip fracture onwards.

**Table 1:** Baseline characteristics of the 18 included studies.

Article	Region	Type of study	Follow-up	Age	n Female	Years studied	n patients	n stroke	Outcomes	MINORS
Atzmon et al. 2018^10^	Israel	Retrospective cohort	9 years	79.3	NS	2003–2014	2195	110	Incidence, hypertension, diabetes, atrial fibrillation, renal insufficiency, gender	16
Goh et al. 2020^11^	UK	Prospective cohort	120 days	≥ 60	NS	2020	8673	52	Incidence	19
Griffin et al. 2015^12^	UK	Retrospective cohort	1 year	83.1	503 (67.9%)	2011	741	1	Incidence	20
Hansson et al. 2015^13^	Sweden	Retrospective cohort	1 year	84.0	482 (72.6%)	2011	664	11	Incidence	17
He et al. 2022^18^	China	Retrospective cohort	90 days	79.2	1295 (51.5%)	2017–2020	2517	63	Incidence , hypertension, diabetes, atrial fibrillation, heart disease, BMI, gender, albumin	19
Kang et al. 2011^19^	Taiwan	Case control in cohort	1 year	63.9	918 (43.7%)	2001–2004	2101	86	Incidence	18
Lawrence et al. 2022^20^	USA	Retrospective cohort	12 years	80.2	7088 (79.4%)	1982–1993	8930	85	Incidence	15
Lowe et al. 2020^21^	USA	Retrospective cohort	7 years	73.3	11,962 (66.3%)	2010–2017	18,042	3181	Incidence	17
de Luise et al. 2007^17^	USA	Retrospective cohort	6 years	80.0	8561 (71.4%)	1998–2003	11,985	2084	Incidence	16
Nho et al. 2014^22^	South Korea	Retrospective cohort	1 year	–	408 (74.5%)	–	548	77	Incidence, gender	12
Pedersen et al. 2017^23^	Denmark	Retrospective cohort	1 year	≥ 55	77,770 (70.3%)	2015	110,563	16,787	Incidence	21
Popa et al. 2009^24^	USA	Retrospective cohort	1 year	80.8	492 (26.1%)	1988–2002	1886	76	Incidence	19
Ramnemark et al. 2015^25^	Sweden	Retrospective cohort	5 years	≥ 65	388 (68.3%)	1980–1997	568	67	Hypertension, diabetes, heart disease, gender	15
Roche et al. 2005^26^	UK	Retrospective cohort	1 year	82	1955 (79.9%)	1999–2003	2448	322	Incidence	16
Rosencher et al. 2005^27^	France	Prospective cohort	Inpatient	82	5183 (75.6%)	2002	6860	48	Incidence	19
Samuel et al. 2017^28^	USA	Retrospective cohort	Inpatient	≥ 65	NS	2011–2012	49,931	221	Incidence	18
Tsai et al. 2015^5^	Taiwan	Retrospective cohort	12 y	72.4	16,920 (71.1%)	1996–2011	23,802	2411	Incidence	18
Yu et al. 2020^29^	China	Retrospective cohort	1 year	80.0	2151 (57.5%)	2014	3743	56	Hypertension, diabetes, heart disease, renal, insufficiency, BMI, gender, albumin	20

Duplicate studies were excluded from this review to avoid overrepresentation of the same data. Case reports and case series were excluded to focus on larger comparative studies with more robust data. Studies with inappropriate designs, such as cross-sectional, case–control, or uncontrolled observational, were also excluded, with a preference for cohort studies. Additionally, studies without clearly defined and separately reported incidences of stroke and hip fractures were excluded to ensure that the included studies provided precise and detailed data regarding the outcomes of interest.

### Information sources

A systematic search of the literature using PubMed, EMBASE, Scopus, and the Cochrane Collaboration Library databases was carried out. No date limits were specified and no language limits were imposed. Studies of interest that appeared in the references of the studies included in the first search were also evaluated.

### Search methods for identification of studies

We used the following search strategy "(hip fracture, stroke, cerebrovascular disease, AND Incidence OR prevalence" (Supplementary File 1). Two reviewers independently agreed on the selection of eligible studies and reached a consensus on which studies to include. All disagreements were resolved by discussion.

### Data extraction and data items

A data extraction form was designed in Excel to extract relevant data for the review. For each study that met the criteria, the following information was extracted: name of the first author, year of publication, study design, study duration, geographical setting, age of participants, treatment received, total sample size, stroke incidence, sex, BMI, and personal history (smoking, hypertension, diabetes, heart disease, atrial fibrillation, and renal failure). Albumin (g/L) was also analyzed as an analytical marker. During the review process, two reviewers independently examined the included studies and performed data extraction using a previously designed extraction form. If any discrepancies or disagreements arose between the two reviewers regarding the interpretation or extraction of data, the following steps were followed to resolve them. Discussion and consensus: The two reviewers discussed the discrepancies and attempted to reach a consensus through dialogue and a joint review of the studies in question. During this stage, differences in interpretation were analyzed, and an agreed-upon solution was sought. Consultation of a third reviewer: If a consensus could not be reached after discussion between the two reviewers, a third reviewer who had not participated in the initial review was consulted. This third reviewer, with relevant experience and knowledge in the field, evaluated the discrepancies and made impartial decisions based on their independent review of the studies and relevant information. Involving a third reviewer ensured the objective and neutral resolution of the discrepancies. It is important to note that a third reviewer was included only when two initial reviewers did not agree. The third reviewer had the authority to make a final decision regarding the extraction of disputed data, thus ensuring a definitive resolution.

### Assessment of risk of bias in included studies

The quality of the included studies was assessed independently by two authors using the Methodological Index for Non-Randomized Studies (MINORS) criteria^15^. It was established to assess the quality of the comparative and non-comparative studies. The maximum score was 24 for the comparative studies and 16 for the non-comparative studies. For non-comparative studies, scores of 0–4 corresponded to very low quality, 5–7 corresponded to low quality, 8–12 corresponded to fair quality, and ≥ 13 corresponded to high quality, respectively. For comparative studies, scores of 0–6 corresponded to very low quality, 7–10 corresponded to low quality, 11–15 corresponded to fair quality, and ≥ 16 corresponded to high quality, respectively (Supplementary Table 1).

### Assessment of results

The Data were analyzed using statistical methods to calculate the standard error (SE) and confidence interval (CI). In cases where SE was not reported, it was estimated based on the prevalence using the following formula: SE = √p (1 − p)/ n; 95% CI = p ± 1.96 × SE^16^, where p represents the prevalence^16^. For categorical variables, odds ratios (ORs) with 95% confidence intervals (CI were computed to assess the likelihood of an event occurring. Continuous variables, such as measurements or scores, were analyzed by calculating the mean difference (MD) along with the 95% CI, enabling comparisons of average values between different groups. Heterogeneity, which indicates variability among study results, was assessed using statistical tests such as the chi-square test and I2 statistic. I2 values ranged from 0 to 100%, with 25%, 50%, and 75% indicating low, moderate, and high heterogeneity, respectively. Different statistical models were employed based on the level of heterogeneity. In the absence of significant heterogeneity, a fixed-effects model was used, assuming that all the studies estimated the same underlying effect. Conversely, in the presence of significant heterogeneity indicating differences between studies, a random-effects model was applied, taking into account these variations. Statistical analysis was conducted using the Review Manager 5.4 software package provided by Cochrane Collaboration.

### Risk of bias across the studies

To evaluate the potential for publication bias, where studies with positive or significant results were more likely to be published, funnel plots were utilized (Review Manager 5.4). These plots visually assess any asymmetry or potential bias in the data.

### Additional analyses

Subgroup analyses were performed to explore potential variations based on geographic region (American, European, or Asian), follow-up time, and mean age. These analyses helped identify whether these factors influenced the relationship between variables. Sensitivity analysis was conducted to assess the robustness of the findings. This involved excluding the study with the highest weight from the comparisons made for all outcomes, thus enabling the evaluation of whether the overall results were influenced by any single study.

## Results

### Study selection

The initial search yielded 635 results that were further refined by excluding review studies and case reports, leaving 470 articles. After removing duplicates and studies that were not of the cohort type (which provided incidence data), the search yielded 120 results, resulting in the elimination of 350 studies. After reviewing the titles and abstracts, an additional 109 studies were excluded because they did not focus on hip fracture or stroke, reported the incidence of hip fracture after stroke, or did not share variables. This process led to the identification of 11 studies that met our inclusion criteria. After reviewing the references of the included articles, 7 additional studies met the inclusion criteria. Finally, 18 studies were included in the meta-analysis (Fig. 1)^5,10–13,17–29^.

### Characteristics of the included studies

Table 1 presents the main characteristics of the included studies. Eighteen studies between 2005 and 2022 were included. All of these studies were cohort studies. Five studies were from the United States, seven from Europe, and six from Asia. The mean age of the patients ranged from 55 to 84 years old. The patients were treated with total hip replacement or osteosynthesis. The follow-up time varied between studies assessing the incidence during admission for up to 12 years. A total of 256,197 patients were included in this study. Regarding quality analysis using the MINORS scale, all studies were comparative; therefore, the maximum score was 24 points. In our analysis, of 15/18 studies were of high quality.

### Outcomes

The overall incidence of stroke after hip fracture was 6.72% (95% CI 4.37% to 9.07%) (Table 2). The incidence varies by region. It was highest in the Americas at 8.09% (95% CI 3.60–12.58%), followed by Asia at 6.21% (95% CI 2.55–9.88%), and Europe at 6.17% (95% CI 1.01–11.34%) (Table 2). However, the differences between the regions were not statistically significant. Looking at the different follow-up periods, the stroke incidence during the initial hospital admission alone was 0.57% (95% CI 0.32–0.82%). For studies assessing stroke incidence at 1 year after hip fracture, it was 5.69% (95% CI 1.14% to 10.24%). For studies with follow-up beyond one year, the stroke incidence was 10.48% (95% CI 3.74–17.23%) (Table 2).

**Table 2:** Summary table of the data from studies included on stroke after hip fracture.

Subgroup	Sample size (n)	n of included studies	Random effect model (incidence 95% CI)	P-value
Overall incidence	256,197	18	6.72% (4.37% to 9.07%)	
Region incidence				
Asia	34,906	6	6.21% (2.55% to 9.88%)	p > 0.05
America	90,774	5	8.09% (3.60% to 12.58%)	
Europe	130,517	7	6.17% (1.01% to 11.34%)	
Follow-up periods				
Inpatient	56,791	2	0.57% (0.32% to 0.82%)	p < 0.05*
1 year follow-up	133,884	10	5.69% (1.14% to 10.24%)	
Until 12 years	65,522	6	10.48% (3.74% to 17.23%)	
Age				
≥ 80 years old	37,257	8	4.94% (2.58% to 7.29%)	p > 0.05
< 80 years old	48,657	5	7.87% (2.43% to 13.31%)	

*Statistically significant.

**Figure 2:**
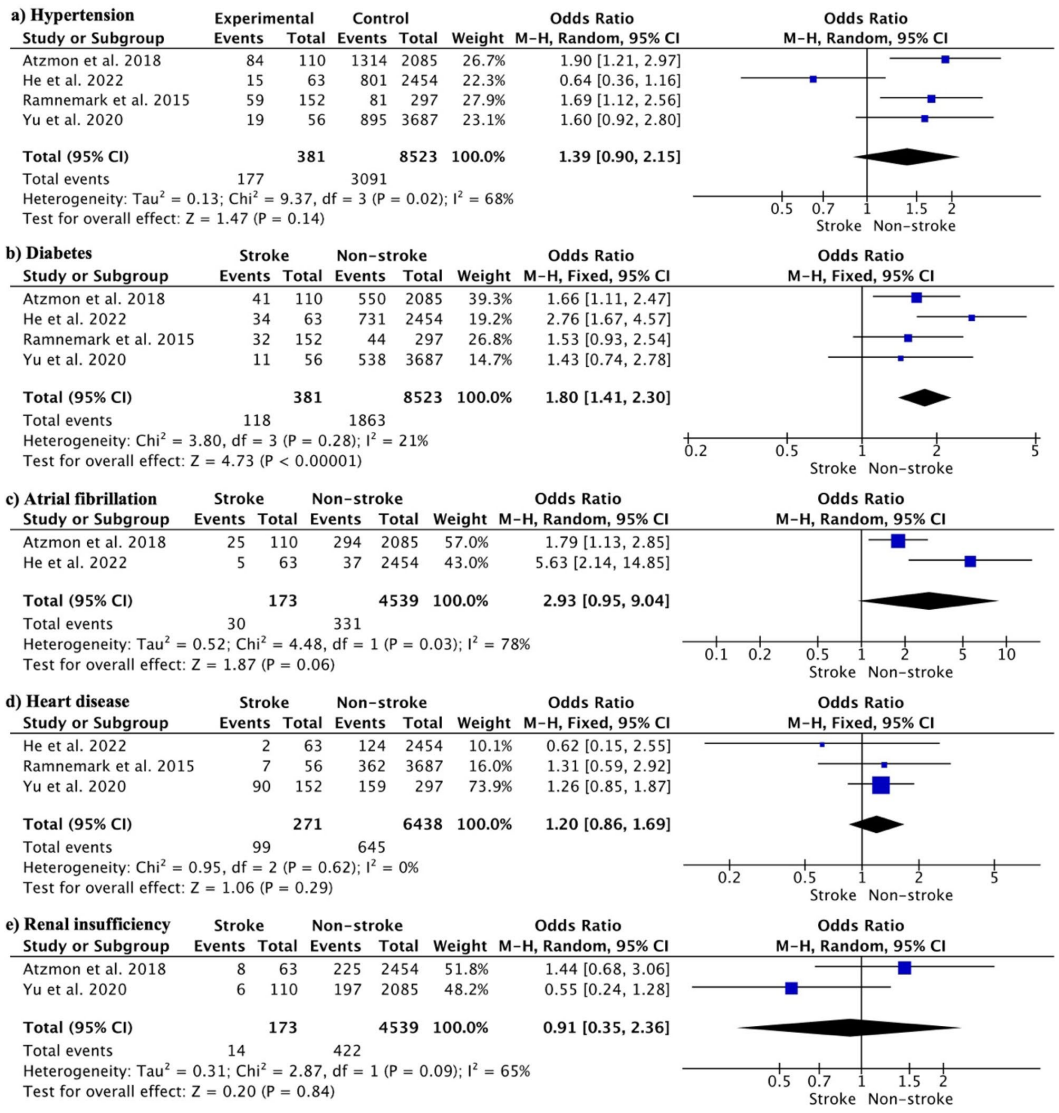
Meta-analysis of the effect of comorbidities on the incidence of stroke after hip fracture: (**a**) hypertension, (**b**) diabetes, (**c**) atrial fibrillation, (**d**) heart disease, and (**e**) renal insufficiency.

In terms of comorbidities, 46.46% of the hip fracture patients who had a stroke also had hypertension. Other comorbidities included diabetes (30.97%), atrial fibrillation (17.34%), heart disease (36.53%) and renal insufficiency (8.09%). Hypertension did not significantly increase the stroke risk (OR 1.39, 95% CI 0.90 to 2.15; p = 0.02) (Fig. 2a). Diabetes significantly increased stroke risk (OR 1.80, 95% CI 1.41 to 2.30; p < 0.00001) (Fig. 2b). Atrial fibrillation (OR 2.93, 95% CI 0.95 to 9.04; p = 0.06) (Fig. 2c), heart disease (OR 1.20, 95% CI 0.86 to 1.69; p = 0.29) (Fig. 2d) and renal insufficiency (OR 0.91, 95% CI 0.35 to 2.36; p = 0.84) (Fig. 2e) was not associated with a significantly higher risk of stroke after hip fracture.

Regarding patient characteristics, a BMI greater than 24.4 did not significantly increase the risk of stroke (OR 1.07, 95% CI 0.74 to 1.56; p = 0.72; participants = 6260; studies = 2; I2 = 0%). In addition, 25.21% of those with stroke smoked compared to 23.37% in the non-stroke group, with no significant differences (OR 1.06, 95% CI 0.69 to 1.61; p = 0.79; participants = 6260; studies = 2; I2 = 48%). The incidence of stroke in women was 58.61% (95% CI 42.67–74.56%), whereas in men, the incidence of stroke was 41.38% (95% CI 25.55–57.21%. There was no significant difference between men and women (OR 1.15, 95% CI 0.91 to 1.46; p = 0.17; participants = 9452; studies = 5; I2 = 20%).

**Figure 3:**
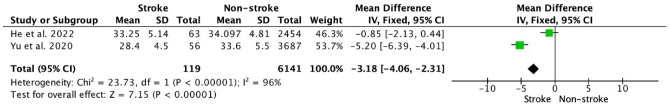
Fixed effects forest plot analyzing the analytic results by albumin. Low albumin levels increased the incidence of stroke after hip fracture (p < 0.001).

With respect to the analytical results, only albumin could be assessed, with low albumin being a risk factor for stroke in patients (MD − 3.18, 95% CI − 4.06 to − 2.31; p < 0.00001; participants = 6260; studies = 2; I2 = 96%) (Fig. 3).

### Additional analysis

**Figure 4:**
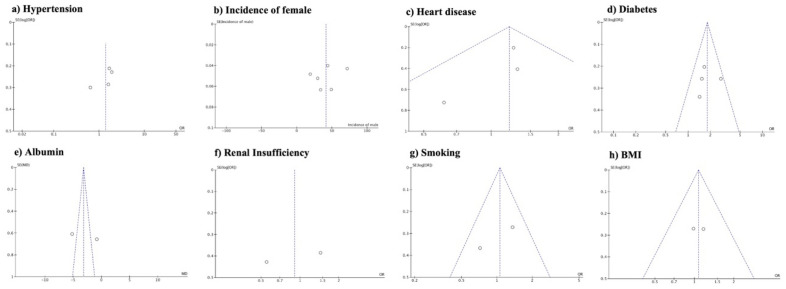
Funnel plot assessment by review manager, showing asymmetry regarding hypertension (**a**), incidence of females among groups (**b**), heart disease (**c**), diabetes (**d**), Albumin (**e**), renal insufficiency (**f**), smoking (**g**) and BMI (**h**).

Funnel plot analysis revealed asymmetry with respect to hypertension, incidence of female sex, albumin level, and renal insufficiency, suggesting a potential publication bias or selective reporting of studies with significant or positive results for these variables. Conversely, no asymmetry was observed regarding heart disease, diabetes, smoking, and BMI, indicating a relatively balanced representation of studies across different levels or categories of these factors, and a lower likelihood of publication bias or selective reporting (Fig. 4). A sensitivity analysis was performed by eliminating the top-weight studies from the comparisons of all outcomes. The direction of the results did not change for any of the examined variables.

## Discussion

This meta-analysis analyzed the overall incidence of stroke after hip fracture and the associated risk factors. The incidence of stroke after hip fracture was 6.72% (number/total number), with the United States being the geographic region with the highest incidence. The incidence during admission was 0.57%; during the first year, it was 5.69%; and in studies that evaluated the incidence at more than 1 year, it was 10.48%. Diabetes is a known risk factor for stroke. Hypertension, heart disease, atrial fibrillation, and renal insufficiency increased the incidence of stroke; however, there were no significant differences between the stroke and non-stroke groups. The incidence in women was higher, but there were no significant differences, and a high BMI did not show significant differences. Smoking was also not a risk factor. Finally, with respect to blood tests, low albumin significantly increased the incidence of stroke.

Stroke is a potential complication in hip fracture patients that increases mortality and morbidity, as well as healthcare expenditure and costs^29^. The incidence of stroke after hip fracture varies across studies, ranging from 0.13%^12^ to 17.63%^21^. Our subgroup analysis showed that the United States had the highest incidence (8.09%), followed by Asia (6.21%) and Europe (6.17%), although there were no significant differences between geographic regions. In the United States, comprehensive stroke prevention programs should be implemented targeting elderly adults, as approximately 80% of strokes are considered preventable with optimal care^30^. In Europe, stroke-related mortality has increased over time, whereas in China, the actual incidence of post-hip fracture stroke has increased dramatically in recent decades^31,32^.

Regarding patient demographic characteristics, female gender was not found to be a statistically significant risk factor in our analysis, with 58.61% of stroke patients being women versus 41.38% men (OR 1.15, 95% CI 0.91 to 1.46)^31^. However, only six of the 18 included studies examined sex, so this finding should be interpreted with caution. Women are generally known to have a higher incidence of stroke across populations^31^. With respect to obesity, defined as a BMI greater than 24.4, there were no significant differences in stroke risk, although only two studies specifically analyzed this outcome. Previous research has shown that high BMI substantially increases mortality in patients with cardiovascular diseases, including stroke^33^.

The results of this meta-analysis have notable clinical implications for the healthcare providers involved in managing patients with hip fractures. The substantial incidence of post-hip fracture stroke (6.72%) highlights the need for diligent monitoring and proactive prevention in this high-risk population. Clinicians should maintain a high index of suspicion for stroke, particularly in the first year following a fracture, based on the peak incidence identified in 1-year follow-up studies. Frequent follow-up visits and neurological assessments are warranted to detect the signs of stroke early. There is value in optimizing modifiable risk factors using a precision medicine approach. For example, diabetes was found to nearly double the stroke risk after hip fracture, with one-third of the stroke patients being diabetic. Aggressive glucose control and hemoglobin A1c monitoring may be useful in patients with hip fractures and diabetes.

Although hypertension, heart disease, atrial fibrillation, and renal insufficiency were not statistically significant predictors of stroke, understanding the nuances of an individual patient's comorbidity profile can still help guide personalized prevention tactics^29^. Low serum albumin levels have emerged as a potential novel biomarker for heightened stroke risk and warrants further research^18^. The incorporation of albumin testing into hip fracture care pathways may be useful in risk stratification.

When comparing the results of this meta-analysis with those of individual primary studies, the only comparisons that could be made were the data presented in the included studies, as no other meta-analyses examining the incidence of stroke after hip fractures were identified. Several factors, including heart disease, renal insufficiency, high BMI, and smoking status, did not demonstrate a statistically significant association with an increased stroke risk in this meta-analysis. The lack of significant effects for these comorbidities and health behaviors could potentially be explained by the limitations in the stratification of data from primary studies. For example, more severe renal dysfunction may confer increased odds of post-hip fracture stroke compared to mild renal impairment. Similarly, the effects of BMI may depend on the category, with obesity Classes II and III potentially carrying a higher stroke risk than overweight status. Smoking intensity and timing could also modify stroke odds, with current heavy smokers possibly facing a greater risk than former or light smokers. The lack of granular and stratified data limits this meta-analysis from detecting subtle or nonlinear effects. In contrast, some individual studies diverged from our meta-analytic findings that there was no significant association between hypertension and post-hip fracture stroke. Atzmon et al.^10^ and Ramnemark et al.^25^ found that hypertension significantly increases the risk of stroke^10,25^. Variability in hypertension prevalence, age distribution, and research settings could explain these disparities. Similarly, Atzmon et al.^10^ and He et al.^18^ identified atrial fibrillation as an independent predictor of post-fracture stroke; however, this meta-analysis found a non-significant trend^10,18^. These discrepancies again highlight that the synthesized data may fail to capture the nuanced effects detectable in higher-quality primary studies.

In individual studies, there were additional risk factors, such as D-dimer or hyperlipidemia^18^. Studies with shorter follow-up periods have established a lower rate of stroke^19,24^. Drugs such as anticoagulants or aspirin could not be analyzed, although these factors are controversial, since the patients who consume them most are those at greatest risk, thus biasing the results^29^. However, the relationship between hip fracture and stroke has been well studied. Stroke increases the probability of instability, falls, and osteoporosis^3^. This is the first study to show that low albumin levels increase the risk of stroke in patients with hip fracture, although the results should be considered with caution due to the low number of included articles. Yu et al.^29^ also demonstrated that red cell distribution increases stroke and mortality. This could not be confirmed in our study, as there were no further articles that included these analytical outcomes^29^.

Regarding conflicts of interest, five studies did not report any, 12 studies reported no conflicts of interest, and one author from a study received research funding from a medical association. In this study, the incidence of stroke was 4.03% and the overall average incidence was 6.72%. The study had a follow-up period of one year, and the overall incidence at one year was 5.69%. Therefore, the incidence in this study did not vary significantly. Regarding funding received, nine studies did not report any, four studies did not receive funding, and five studies reported receiving funding. These five studies had a combined incidence of 6.29%, which is very similar to those that did not receive funding (6.88%). Four of the five studies had a one-year follow-up, showing an incidence of 5.33%, similar to those that did not receive funding (5.93%). In terms of risk factors, the study with funding showed significant differences compared to the study that did not receive funding, although significant differences were observed overall. There were no differences between the studies that received funding regarding hypertension, BMI, atrial fibrillation, and renal insufficiency. However, regarding diabetes, the study that received funding did not show an association with a higher incidence of stroke than the studies that did not receive funding, which showed an association with a higher incidence. Conflicts of interest and funding did not significantly influence the results of this study (Supplementary Table 2).

This study had several limitations that could affect the generalizability of the conclusions. First, the inclusion of studies that did not specify whether hip fractures underwent surgery introduced variability in outcomes, potentially impacting the applicability of the findings to specific subgroups. The lack of consideration of the specific type of fracture (intracapsular or extracapsular) and the types of interventions performed (e.g., total hip arthroplasty or osteosynthesis) limits the ability to assess the influence of these factors on stroke incidence, potentially limiting the generalizability of the conclusions to different fracture and treatment scenarios. Variations in follow-up duration, although addressed through subgroup analysis, may still introduce bias and affect the comparability of the results across different studies. Moreover, the absence of information regarding the type of stroke (hemorrhagic or ischemic) and anesthesia hampers the assessment of their potential impact on stroke incidence and the generalizability of the findings to different stroke subtypes and anesthesia protocols. The inability to stratify the data using the ASA scale further limits our understanding of the relationship between stroke and patients' overall health status. Some studies focusing on complications rather than stroke alone may have resulted in limited extracted information specifically related to stroke incidence, potentially affecting the generalizability of the conclusions to the stroke population. It should be noted that Review Manager was the sole software used for analysis in this review, and formal tests for publication bias, such as Egger's test, could not be conducted. Survival analysis based on a small number of heterogeneous studies may not allow robust comparisons or general conclusions. Additionally, it is important to acknowledge the retrospective nature of the majority of the included studies and their reporting of variables, such as age, using means or medians, which could introduce biases and affect the generalizability of the conclusions to different study designs and age distributions.

However, this meta-analysis had certain limitations owing to potential sources of bias in the included studies. The asymmetry detected in the funnel plots for some variables indicated that publication bias likely affected the pooled estimates. Specifically, the selective reporting of studies with statistically significant associations between stroke and hypertension, female sex, low albumin levels, and renal insufficiency may have led to an overestimation of these effects. Furthermore, substantial statistical heterogeneity was observed in some analyses, including atrial fibrillation and renal insufficiency, as evidenced by high I2 values. This heterogeneity suggests variability across studies in factors, such as patient demographics, fracture types, and healthcare settings. The differing adjustments for confounders across studies may have contributed to heterogeneity. Although no major changes resulted from the sensitivity analyses, these sources of between-study variability should be considered when interpreting the pooled findings, especially for analyses with high heterogeneity.

Future studies should consider incorporating novel markers, such as vitamin D^34^, and providing direct analytical results to assess biomarkers specifically related to stroke incidence, thereby enhancing the generalizability of the findings and allowing for a more comprehensive understanding of the topic.

On the other hand, some of the strengths of this meta-analysis were that it is the first meta-analysis to analyze the incidence and risk factors for stroke after hip fracture, and we included a large sample size, which is interesting for the estimation of the overall incidence.

## Conclusion

The incidence of stroke after a hip fracture was 6.72%. The incidence of stroke increases over time, and the associated risk factors include diabetes and low albumin levels. Multidisciplinary treatment between different teams of orthopedics, rehabilitation, and specialties, including comorbidities such as endocrine, internal medicine, and cardiology, is recommended. This meta-analysis highlights the need for additional high-quality studies to elucidate the epidemiology and risk factors of post-hip fracture stroke, especially in regions outside the United States. Future research should enroll larger multicountry patient cohorts with extensive data collection on potential demographic, clinical, and biochemical predictors. There is a particular need to better understand how antiplatelet/anticoagulant use, atrial fibrillation, renal disease, and diabetes management influence the stroke risk after hip fractures. Studies with longer-term follow-up could provide valuable data on stroke incidence beyond one year post-fracture. The effects of elevated levels of inflammatory markers, coagulation factors, and lipid profiles warrant further investigation. Genetic and miRNA biomarkers associated with post-fracture susceptibility to stroke should be explored. Future studies must carefully consider confounding factors and biases that could distort the observed associations.

### Supplementary Information

Supplementary Information 1.

Supplementary Table S1.

Supplementary Table S2.

Supplementary Table S3.

## Data Availability

The datasets used and/or analysed during the current study available from the corresponding author on reasonable request.
