# Supplementary material for: Incidence and risk factors for stroke after hip fracture: a meta-analysis
Source: Sci Rep. 2023 Oct 17;13:17618. doi: 10.1038/s41598-023-44917-7 (PMC10582073; doi:10.1038/s41598-023-44917-7)
Supplement: Supplementary file 1 — Supplementary Information 1. [file 41598_2023_44917_MOESM1_ESM.docx]

**Supplementary File 1.** Pubmed search strategy.

Search: **("hip fracture") AND (stroke OR cerebrovascular) AND (Incidence OR prevalence)**

"hip fracture"[All Fields] AND ("stroke"[MeSH Terms] OR "stroke"[All Fields] OR "strokes"[All Fields] OR "stroke s"[All Fields] OR "cerebrovascular"[All Fields]) AND ("epidemiology"[MeSH Subheading] OR "epidemiology"[All Fields] OR "incidence"[All Fields] OR "incidence"[MeSH Terms] OR "incidences"[All Fields] OR "incident"[All Fields] OR "incidents"[All Fields] OR ("epidemiology"[MeSH Subheading] OR "epidemiology"[All Fields] OR "prevalence"[All Fields] OR "prevalence"[MeSH Terms] OR "prevalance"[All Fields] OR "prevalences"[All Fields] OR "prevalence s"[All Fields] OR "prevalent"[All Fields] OR "prevalently"[All Fields] OR "prevalents"[All Fields]))

**Translations**

**stroke:** "stroke"[MeSH Terms] OR "stroke"[All Fields] OR "strokes"[All Fields] OR "stroke's"[All Fields]

**Incidence:** "epidemiology"[Subheading] OR "epidemiology"[All Fields] OR "incidence"[All Fields] OR "incidence"[MeSH Terms] OR "incidences"[All Fields] OR "incident"[All Fields] OR "incidents"[All Fields]

**prevalence:** "epidemiology"[Subheading] OR "epidemiology"[All Fields] OR "prevalence"[All Fields] OR "prevalence"[MeSH Terms] OR "prevalance"[All Fields] OR "prevalences"[All Fields] OR "prevalence's"[All Fields] OR "prevalent"[All Fields] OR "prevalently"[All Fields] OR "prevalents"[All Fields]
