# Supplementary material for: Incidence and risk factors for stroke after hip fracture: a meta-analysis
Source: Sci Rep. 2023 Oct 17;13:17618. doi: 10.1038/s41598-023-44917-7 (PMC10582073; doi:10.1038/s41598-023-44917-7)
Supplement: Supplementary file 2 — Supplementary Table S1. [file 41598_2023_44917_MOESM2_ESM.docx]

**Supplementary Table 1.** Assessment of the quality of studies through Methodological Index for Non-Randomized Studies (MINORS).

| **Study** | **Clearly stated aim** | **Consecutive patients** | **Prospective collection data** | **Endpoints** | **Assessment endpoint** | **Follow-up period** | **Loss less than 5%** | **Study size** | **Adequate control group** | **Contemporary group** | **Baseline control** | **Statistical analyses** | **MINORS** |
| --- | --- | --- | --- | --- | --- | --- | --- | --- | --- | --- | --- | --- | --- |
| **Atzmon et al. 2018^10^** | 2 | 2 | 0 | 2 | 2 | 2 | 0 | 2 | 0 | 2 | 0 | 2 | 16 |
| **Goh et al. 2020^11^** | 2 | 2 | 2 | 2 | 2 | 1 | 0 | 2 | 1 | 2 | 1 | 2 | 19 |
| **Griffin et al. 2015^12^** | 1 | 2 | 2 | 2 | 2 | 2 | 0 | 1 | 2 | 2 | 2 | 2 | 20 |
| **Hansson et al. 2015^13^** | 2 | 2 | 0 | 2 | 2 | 2 | 1 | 1 | 1 | 2 | 0 | 2 | 17 |
| **He et al. 2022^18^** | 2 | 2 | 0 | 2 | 2 | 2 | 0 | 2 | 2 | 2 | 1 | 2 | 19 |
| **Kang et al. 2011^19^** |  |  |  |  |  |  |  |  |  |  |  |  | 18 |
| **Lawrence et al. 2022^20^** | 2 | 2 | 0 | 2 | 1 | 2 | 0 | 2 | 0 | 2 | 0 | 2 | 15 |
| **Lowe et al. 2020^21^** | 2 | 2 | 0 | 2 | 2 | 2 | 0 | 2 | 1 | 2 | 0 | 2 | 17 |
| **de Luise et al. 2007^17^** | 2 | 2 | 0 | 2 | 2 | 2 | 0 | 2 | 0 | 2 | 0 | 2 | 16 |
| **Nho et al. 2014^22^** | 1 | 2 | 0 | 2 | 2 | 2 | 0 | 1 | 0 | 0 | 0 | 2 | 12 |
| **Pedersen et al. 2017^23^** | 1 | 2 | 2 | 2 | 2 | 2 | 0 | 2 | 2 | 2 | 2 | 2 | 21 |
| **Popa et al. 2009^24^** | 2 | 2 | 0 | 2 | 2 | 2 | 2 | 2 | 1 | 2 | 0 | 2 | 19 |
| **Ramnemark et al. 2015^25^** | 2 | 2 | 0 | 2 | 2 | 2 | 0 | 1 | 1 | 2 | 1 | 0 | 15 |
| **Roche et al. 2005^26^** | 2 | 2 | 0 | 2 | 2 | 2 | 0 | 2 | 0 | 2 | 0 | 2 | 16 |
| **Rosencher et al. 2005^27^** | 2 | 2 | 2 | 2 | 2 | 2 | 0 | 1 | 1 | 2 | 1 | 2 | 19 |
| **Samuel et al. 2017^28^** | 2 | 2 | 0 | 2 | 2 | 2 | 0 | 2 | 1 | 2 | 1 | 2 | 18 |
| **Tsai et al. 2015^5^** | 1 | 2 | 0 | 2 | 2 | 2 | 0 | 2 | 1 | 2 | 2 | 2 | 18 |
| **Yu et al. 2020^29^** | 2 | 2 | 0 | 2 | 2 | 2 | 0 | 2 | 2 | 2 | 2 | 2 | 20 |
