# Supplementary material for: Incidence and risk factors for stroke after hip fracture: a meta-analysis
Source: Sci Rep. 2023 Oct 17;13:17618. doi: 10.1038/s41598-023-44917-7 (PMC10582073; doi:10.1038/s41598-023-44917-7)
Supplement: Supplementary file 3 — Supplementary Table S2. [file 41598_2023_44917_MOESM3_ESM.docx]

**Supplemnetary Table 2.** Conflict of interests and funding.

| **Study** | **COI** | **Funding** |
| --- | --- | --- |
| **Atzmon et al. 2018^10^** | No | NR |
| **Goh et al. 2020^11^** | NR | NR |
| **Griffin et al. 2015^12^** | No | Yes^2^ |
| **Hansson et al. 2015^13^** | NR | NR |
| **He et al. 2022^18^** | No | No |
| **Kang et al. 2011^19^** | No | No |
| **Lawrence et al. 2022^20^** | NR | NR |
| **Lowe et al. 2020^21^** | No | No |
| **de Luise et al. 2007^17^** | No | NR |
| **Nho et al. 2014^22^** | No | NR |
| **Pedersen et al. 2017^23^** | No | Yes^3^ |
| **Popa et al. 2009^24^** | Yes^4^ | Yes^4^ |
| **Ramnemark et al. 2015^25^** | NR | NR |
| **Roche et al. 2005^26^** | No | No |
| **Rosencher et al. 2005^27^** | No | NR |
| **Samuel et al. 2017^28^** | NR | NR |
| **Tsai et al. 2015^5^** | No | Yes^5^ |
| **Yu et al. 2020^29^** | No | Yes^1^ |

NR: Not reported.

^1^The study was funded by 3-3-3 talent project for high-level talents of Hebei Province.

^2^The WHiTE study is funded by the NIHR Oxford Biomedical Research Centre. WHiTE4 is funded by X-BOLT Ltd.

^3^The study was supported by a grant from the Aarhus University Research Foundation and by the Program for Clinical Research Infrastructure (PROCRIN) established by the Lundbeck Foundationand the Novo Nordisk Foundation.

^4^One author discloses funding from the American Heart Association.

^5^This study is supported in part by the Clinical Trialand Research Center of Excellence, Taiwan Ministry of Health and Welfare.
